# Diagnostic accuracy of gastric filling ultrasound combined with BMI for gastroesophageal reflux disease

**DOI:** 10.3389/fmed.2026.1727623

**Published:** 2026-02-19

**Authors:** Feng Tang, Huizhen Yu, Tianjun Zhao, Lin Lin, Peiwen Dong, Kaidi Sun, Xiaobin Sun, Qiong Wang

**Affiliations:** 1 Department of Gastroenterology, The Affiliated Hospital of Southwest Jiaotong University, The Third People's Hospital of Chengdu, Chengdu, China; 2 Department of Ultrasound Medicine, The Affiliated Hospital of Southwest Jiaotong University, The Third People's Hospital of Chengdu, Chengdu, China; 3 Department of Cardiology, The Affiliated Hospital of Southwest Jiaotong University, The Third People's Hospital of Chengdu, Chengdu, China

**Keywords:** 24-h pH impedance-monitoring, BMI, diagnostic accuracy, gastric filling ultrasound, gastroesophageal reflux disease

## Abstract

**Purpose:**

This study aimed to evaluate the diagnostic accuracy of gastric filling ultrasound, in combination with body mass index (BMI), for the dynamic assessment of gastroesophageal reflux in patients with gastroesophageal reflux disease (GERD)-like symptoms, using 24-h pH impedance monitoring and esophagogastroduodenoscopy (EGD) as reference standards.

**Methods:**

We enrolled 155 patients with GERD-like symptoms in our hospital. The results of gastric filling ultrasound, combined with BMI, were compared with 24-h pH impedance monitoring and EGD to assess GERD. Diagnostic accuracy was assessed using sensitivity, specificity, positive predictive value (PPV), negative predictive value (NPV), and Cohen’s kappa statistics. A calibration curve was constructed to assess agreement between the model’s predicted probabilities and the observed outcomes. Decision curve analysis (DCA) was performed to evaluate clinical utility.

**Results:**

GERD patients exhibited significant differences in terms of age, BMI, His angle, and all reflux-related metrics (*p* < 0.05). The BMI-based gastric filling ultrasound composite score demonstrated an area under the curve of 0.826 with an optimal cut-off of 4.50. Using this threshold, scores 6–8 were defined as GERD; sensitivity was 83.75%, specificity was 65.33%, PPV was 72.04%, NPV was 79.03%, and the overall accuracy was 74.84%. The observed agreement was 0.75, the expected agreement was 0.50, and the kappa was 0.494. The calibration curve indicated good agreement between the model’s predicted probabilities and the observed outcomes, with a mean absolute error of 0.034. Decision curve analysis (DCA) demonstrated that the model provided a superior net benefit over both the “treat-all” and “treat-none” strategies across a wide range of clinically relevant threshold probabilities (approximately 10–65%).

**Conclusion:**

Gastric filling ultrasound is a dynamic imaging technique that visualizes GERD symptoms. The BMI-based gastric filling ultrasound, given its good correlation with 24-h pH impedance monitoring, is a promising auxiliary diagnostic tool for GERD.

## Introduction

Gastroesophageal reflux disease (GERD) is a common digestive disorder characterized by heterogeneous symptomatology and multiple pathogenic mechanisms. Acid reflux and heartburn are typical symptoms. Atypical symptoms include chronic cough, asthma, hoarseness, chronic laryngitis, chest pain, indigestion, and nausea (1). According to the Global Burden of Disease 2019 study, the number of GERD cases in 2019 was 784.00 million, reflecting a 77.50% increase from 1990 (2). The global prevalence of GERD varies widely, ranging from 2.50 to 51.20% across populations, with an average prevalence of 14.80%. Notably, significant regional heterogeneity exists, with substantially higher prevalence rates in Western countries compared to Asian countries (3). However, with rising economic development and lifestyle-related pressure, the prevalence of GERD in Asia has been increasing year by year, and the prevalence is approximately 7.70% in China (4). GERD substantially impairs quality of life and reduces work productivity, generating considerable disease-related economic burden (5). In addition, GERD is associated with serious complications, including Barrett’s esophagus, esophageal stricture, and esophageal adenocarcinoma.

The accurate diagnosis of GERD is foundational to optimizing therapeutic outcomes and preventing complications. Contemporary guidelines, including the Lyon Consensus 2.0 and the American Gastroenterological Association (AGA) 2022 Clinical Practice Update, recommend a strategy based on objective testing to confirm the diagnosis of GERD (6, 7). This approach prioritizes the identification of conclusive evidence of reflux disease, primarily through esophagogastroduodenoscopy (EGD) and/or ambulatory reflux monitoring. According to the Lyon Consensus 2.0, conclusive evidence on EGD includes Los Angeles grade B or greater esophagitis, Barrett’s esophagus, or peptic stricture. When EGD fails to provide a diagnosis, ambulatory reflux monitoring—particularly prolonged wireless pH monitoring without proton pump inhibitor therapy—is the preferred test to quantify pathologic acid exposure, with an acid exposure time (AET) > 6.00% being diagnostic of GERD (6). The AGA guidelines further reinforce this objective, phenotype-driven strategy, particularly in patients with inadequate response to an empiric proton pump inhibitor (PPI) trial or those presenting with atypical symptoms (7). The 24-h pH impedance monitoring can not only monitor reflux but also detect the relationship between reflux degree, symptoms, and posture (8). However, the clinical utility of 24-h pH impedance monitoring is constrained by several inherent limitations, including its technical complexity, substantial financial cost, prolonged procedural duration, and inability to provide direct anatomical visualization of defects such as hiatal hernias. The placement of a trans-nasal catheter over 24 h may lead to pharyngeal discomfort, nose pain, or rhinorrhea in over 50% of patients, with approximately 6% being unable to tolerate the examination (9). Moreover, EGD is a cornerstone diagnostic modality for GERD in clinical practice. The invasive nature of the procedure often leads to patient discomfort, pain, or intolerance, thereby limiting its feasibility in a subset of individuals (10). Furthermore, a substantial proportion of GERD patients present with non-erosive reflux disease, in which standard EGD often yields false-negative results due to the absence of visible mucosal lesions. It significantly compromises the diagnostic accuracy of GERD. The limitations highlight a critical need for more accessible, well-tolerated, and non-invasive diagnostic tools. Therefore, it is crucial to develop a new non-invasive modality for diagnosing and monitoring GERD.

With contemporary advancements in ultrasonic imaging resolution and ongoing technological refinements, gastric filling ultrasound has been established as a well-tolerated imaging modality characterized by operational simplicity and non-invasiveness, consequently enhancing patient compliance (11). Zhang et al. (12) reported that gastric filling ultrasound has high diagnostic accuracy for peptic ulcer in children, suggesting its potential as a preliminary examination method. Furthermore, Wang et al. (13) researched that gastric filling ultrasound is a useful diagnostic method for preoperative Borrmann classification of gastric cancer. Previous studies have shown that sonographic assessment of findings, such as abdominal esophageal length, esophageal wall thickness, and His angle, provides important diagnostic indicators of reflux and is related to the degree of GERD (14). Widening of His angle and shortening of the abdominal esophagus are known structural alterations associated with GERD (15, 16). These structural alterations can directly observe and quantify the passage of gastric contents, such as the echoic contrast agent, into the esophagus, documenting the frequency, duration, and width of reflux episodes. This technique provides direct functional evidence of reflux, similar to the principle of barium studies but with greater sensitivity and without radiation. The value of sonography in reflux assessment is further highlighted by Jang et al., who concluded that color Doppler sonography was highly sensitive and easier to use than pH monitoring for diagnosing GERD in children (17). These findings collectively support the exploration of gastric filling ultrasound as a viable tool for assessing GERD in adult populations. Separately, numerous studies have reported a significant association between elevated body mass index (BMI) and GERD. Maleki et al. (18) revealed that the prevalence of GERD was significantly high in individuals with higher BMI. Sudipta et al. (19) also showed that BMI ≥ 25.0 was independently associated with GERD in southern India.

Currently, there are no studies on the simultaneous use of 24-h pH impedance monitoring, EGD, and gastric filling ultrasound in GERD. Our study explores the potential of gastric filling ultrasound combined with BMI as a novel, dynamic, and non-invasive auxiliary diagnostic method.

## Materials and methods

### Study population

This study received approval from the local ethics board and was conducted in accordance with the Declaration of Helsinki in its most recent version. Written informed consent was provided by all participants before the examination. Inclusion criteria for this study were as follows: patients with typical GERD-like symptoms such as reflux, acid reflux, or heartburn for at least 6 months, recruited from both the gastroenterology outpatient clinic and the inpatient department (Department of Gastroenterology, Chengdu Third People’s Hospital, Affiliated Hospital of Southwest Jiaotong University, China). The inpatients were admitted specifically for a comprehensive diagnostic workup, which included 24-h pH impedance monitoring, EGD, and gastric filling ultrasound. Patients admitted for other acute medical or surgical conditions were excluded. Clinical data from 155 suspected GERD patients diagnosed between July 2023 and March 2025 were analyzed. Diagnostic criteria: the diagnosis was confirmed by 24-h pH impedance monitoring and/or EGD in accordance with the diagnostic criteria for gastroesophageal reflux. Patients were definitively diagnosed with GERD if they met any of the following criteria based on the reference standards: an acid exposure time (AET) > 6.00% on 24-h pH impedance monitoring, and/or the presence of reflux-related mucosal breaks (erosive esophagitis) or Barrett’s esophagus on EGD.

The inclusion criteria for patients are as follows: (1) age≥18 years; (2) at least one of the following clinical manifestations within the last 4 weeks: reflux, acid reflux, or heartburn. The exclusion criteria of patients are as follows: Patients with (1) dysphagia like achalasia, unintentional weight loss, and other symptoms such as gastrointestinal bleeding, anemia, persistent vomiting, or a palpable abdominal mass; (2) on PPI treatment during 24-h pH impedance monitoring; and (3) accompanied by severe heart, liver, kidney, lung, and other systemic diseases. Patients who underwent gastric filling ultrasound, 24-h pH impedance monitoring, and EGD were also included in this study.

### 24-h pH impedance monitoring

A 24-h pH impedance monitoring was performed using a multichannel pH impedance catheter equipped with six impedance segments and one pH-measuring electrode (Given Imaging, Inc., Ltd.). All subjects underwent trans-nasal placement of a pH impedance-monitoring catheter with the sensor positioned 5 cm above the lower esophageal sphincter (LES), as confirmed by high-resolution manometry. Continuous 24-h pH data were acquired using a portable digital recorder. All data were uploaded and analyzed using AccuView (version 5.2) software (Given Imaging, Inc., Ltd.) according to the Lyon Consensus criteria 2.0 (6). A distal esophageal acid exposure time (AET) > 6.00% off PPI on ambulatory pH monitoring is diagnostic of GERD. The parameters of total reflux episodes >80/day and the DeMeester score were considered supportive evidence for a comprehensive reflux profile; they were not used as independent diagnostic criteria. The DeMeester score is automatically calculated by the analysis software. The parameters contained in the DeMeester score are: total number of reflux episodes, number of episodes longer than 5 min, the duration of the longest reflux episode, total percentage of time of pH < 4 for the total monitoring, and the percentage of time with pH < 4 in an upright position and supine position, respectively (20).

### Gastric filling ultrasound

The gastric filling ultrasound diagnostic instrument uses a 4D convex array probe from Siemens GE Company, with a frequency range of 3.5–5.5 MHz. Patients were instructed to fast for 8–12 h before testing and to take 500 mL of gastric US support agent orally 5 min before testing. The patients were advised to sit, dynamically scan the end of the esophagus, cardia, stomach, and duodenum, and observe whether there are changes in gastric wall morphology, peristaltic frequency, content emptying, reflux, echotexture, and the nature and distribution of pathological blood vessels. Then, the patients were assisted in assuming a supine position. The subxiphoid left costal margin was selected as the scanning plane. The patients were instructed to inhale deeply and hold their breath. The probe was directed toward the left lobe of the liver, and gentle pressure was applied on the abdominal wall. His angle is formed between the esophageal hiatus and the tangential plane of the gastric fundus. The length of the abdominal segment of the esophagus is defined as the distance between the esophageal hiatus and the tangential plane of the gastric fundus. The duration and frequency of gastroesophageal reflux was observed within 5 min and evaluated pathological and physiological reflux. (1) Physiological reflux: no clinical manifestation of gastroesophageal reflux, the frequency ≤1 day/week with mild symptoms, and the duration of reflux ≤2 s, with a frequency of ≤2 times within 5 min. (2) Pathological reflux: the patient has typical symptoms of reflux, with a frequency of more than 1 day per week, severe symptoms, and a reflux duration of ≥3 s within 5 min, with a frequency of ≥3 times (21). The width of the reflux beam is defined as the width at the time of reflux. The images from the gastric filling ultrasound evaluation are presented in a previous study (14).

### Statistical analysis

Continuous variables were presented as medians and interquartile ranges (IQRs), and categorical variables were expressed as absolute values and percentages. A standard 2 × 2 table method was used to calculate diagnostic performance. Sensitivity was defined as the number of true positives divided by the sum of true positives and false negatives; specificity was defined as the number of true negatives divided by the sum of true negatives and false positives. Positive predictive value (PPV) was defined as the number of true positives divided by the sum of true positives and false positives. A composite score was developed by integrating BMI and gastric filling ultrasound parameters. The discriminatory ability of this continuous BMI-based gastric filling ultrasound composite score for predicting GERD was assessed using receiver operating characteristic (ROC) curve analysis. The area under the ROC curve (AUC) was calculated to quantify the model’s overall discrimination performance. A calibration curve was constructed to assess the model’s agreement between the model’s predicted probabilities and the observed outcomes. Decision curve analysis (DCA) was performed to evaluate the model’s clinical utility by quantifying net benefit across a range of threshold probabilities. The diagnostic agreement in identifying GERD among 24-h pH impedance monitoring, EGD, and gastric filling ultrasound was calculated using Cohen’s kappa statistics. For Cohen’s kappa, values of 0.01–0.20 were defined as no agreement, 0.21–0.40 were defined as minimal agreement, 0.41–0.60 were defined as moderate agreement, 0.61–0.80 were defined as substantial agreement, 0.81–0.90 were defined as strong agreement, and >0.90 were defined as almost perfect agreement (22). A *p*-value <0.05 was considered statistically significant.

## Results

### Study population

Of the 158 eligible patients, 155 patients fulfilled the inclusion criteria. All enrolled patients with GERD-like symptoms underwent both high-resolution manometry and 24-h pH impedance monitoring. Three patients were excluded due to a diagnosis of achalasia based on high-resolution manometry. The median patient age was 61 years (IQR: 53.0–67.0 years). Gender distribution was relatively balanced, with 85 female patients (54.84%) and 70 male patients (45.16%). The median BMI was 23.6 (IQR: 23.0–25.4), and the GERD patients had a significantly higher BMI (median: 24.0, IQR: 22.8–25.9) compared to non-GERD subjects (median: 22.7, IQR: 20.4–24.3) (*p* < 0.001). The study population was summarized in Table 1.

**Table 1:** Baseline characteristics of included patients.

Variables	Total	GERD	Non-GERD	*p*-value
No. subjects	155	94	61	
Age	61.0 (53.0–67.0)	62.5 (54.0–68.0)	60.0 (51.5–67.0)	0.212
Gender				0.067
Female	85 (54.84%)	46 (48.90%)	39 (63.90%)	
Male	70 (45.16%)	48 (51.10%)	22 (36.10%)	
BMI index (kg/m^2^)	23.6 (22.0–25.4)	24.0 (22.8–25.9)	22.7 (20.4–24.3)	<0.001
Length of abdominal esophagus (cm)	2.60 (2.30–3.00)	2.60 (2.40–3.00)	2.80 (2.20–3.15)	0.367
Thickness of abdominal esophageal wall (cm)	0.33 (0.30–0.40)	0.35 (0.30–0.40)	0.31 (0.29–0.40)	0.062
Thickness of cervical esophageal wall (cm)	0.20 (0.16–0.22)	0.20 (0.17–0.22)	0.19 (0.15–0.22)	0.095
His angle (°)	92.0 (83.0–101.0)	96.0 (89.0–112.0)	85.0 (75.5–94.0)	<0.001
Duration of reflux in 5 min	8.0 (0.0–17.3)	15.0 (3.0–20.0)	2.0 (0.0–9.0)	<0.001
Number of refluxes in 5 min	1 (0–3)	2 (1–3)	0 (0–2)	<0.001
Width of reflux beam (cm)	1.00 (0.00–1.50)	1.20 (0.70–1.60)	0.30 (0.00–1.20)	<0.001

Diagnosis of gastroesophageal reflux disease based on Lyon Consensus 2.0 statements. Continuous variables are provided as median and interquartile ranges (IQR).

### Gastric filling ultrasound

Gastric filling ultrasound was successfully completed in all 155 patients without any adverse events. The average acquisition time for a gastric filling ultrasound was 5 min. The length of the abdominal esophagus (*p* = 0.367), the thickness of the abdominal esophageal wall (*p* = 0.062), and the thickness of the cervical esophageal wall (*p* = 0.095) showed no difference. His angle (median 96.0 vs. 85.0, *p* < 0.001), duration of reflux (median 15.0 s vs. 2.0 s, *p* < 0.001), number of refluxes (median 2 vs. 0, *p* < 0.001), and width of reflux beam (median 1.20 vs. 0.30, *p* < 0.001) were statistically higher in GERD patients compared to non-GERD patients. The study showed that GERD patients exhibited statistically significant differences in BMI, His angle, and reflux-related metrics (*p* < 0.05) in Table 1.

### Comparison of predictive values and diagnostic agreement

As listed in Table 2, the result of 24-h pH impedance monitoring as diagnostic criteria, the sensitivity of EGD was 60.00%, specificity was 81.33%, PPV was 74.42%, negative predictive value (NPV) was 65.59%, and accuracy was 70.32%. Diagnostic agreement analysis was performed using Cohen’s kappa coefficient to assess inter-rater reliability. The observed agreement was 0.70, the expected agreement was 0.50, and the kappa was 0.41. In Table 3, the sensitivity of gastric filling ultrasound was 81.25%, the specificity was 54.67%, the PPV was 65.66%, the NPV was 73.21%, and the accuracy was 68.39%. The observed agreement was 0.68, the expected agreement was 0.50, and the kappa was 0.36. Compared to EGD as reference, in Table 4, the sensitivity of gastric filling ultrasound was 75.81% and specificity was 44.09%, the PPV was 47.47%, the NPV was 73.21%, and the accuracy was 56.77%. The observed agreement was 0.57, the expected agreement was 0.47, and the kappa was 0.18.

**Table 2:** Comparison of the results of 24-h pH monitoring (reference standard of diagnosis) and gastroscopy.

Method	24-h pH monitoring – pathological	24-h pH monitoring – normal	Total	*p*-value	Kappa index
Gastroscope – pathological	48	14	62	<0.001	0.41
Gastroscope – normal	32	61	93		
Total	80	75	155		

**Table 3:** Comparison of the results of 24-h pH monitoring (reference standard of diagnosis) and gastric filling ultrasound.

Method	24-h pH monitoring – pathological	24-h pH monitoring – normal	Total	*p*-value	Kappa index
Gastric filling ultrasound – pathological	65	34	99	<0.001	0.36
Gastric filling ultrasound – normal	15	41	56		
Total	80	75	155		

**Table 4:** Comparison of the results of the gastroscope (reference standard of diagnosis) and gastric filling ultrasound.

Method	Gastroscope – pathological	Gastroscope – normal	Total	*p*-value	Kappa index
Gastric filling ultrasound – pathological	47	52	99	0.012	0.18
Gastric filling ultrasound – normal	15	41	56		
Total	62	93	155		

### Correlation between ultrasound parameters and 24-h pH impedance monitoring parameters

As shown in Supplementary Table S1, among the various gastric filling ultrasound parameters assessed, only the thickness of the cervical esophageal wall showed statistically significant, positive correlations with the three 24-h pH-monitoring parameters (*p* < 0.05). Specifically, the correlations were moderate for the number of reflux episodes (*r* = 0.302, *p* = 0.009) and AET (*r* = 0.331, *p* = 0.013) and weaker for the DeMeester score (*r* = 0.236, *p* = 0.029). The His angle showed a weak but significant positive correlation solely with the DeMeester score (*r* = 0.217, *p* = 0.044). No other ultrasound parameters, including the length of the abdominal esophagus, thickness of the abdominal esophageal wall, duration and number of reflux events during the procedure, and width of the reflux beam, showed any significant correlation with the pH-monitoring metrics (all *p* > 0.05).

### Diagnostic performance analysis and clinical utility assessment

**Figure 1:**
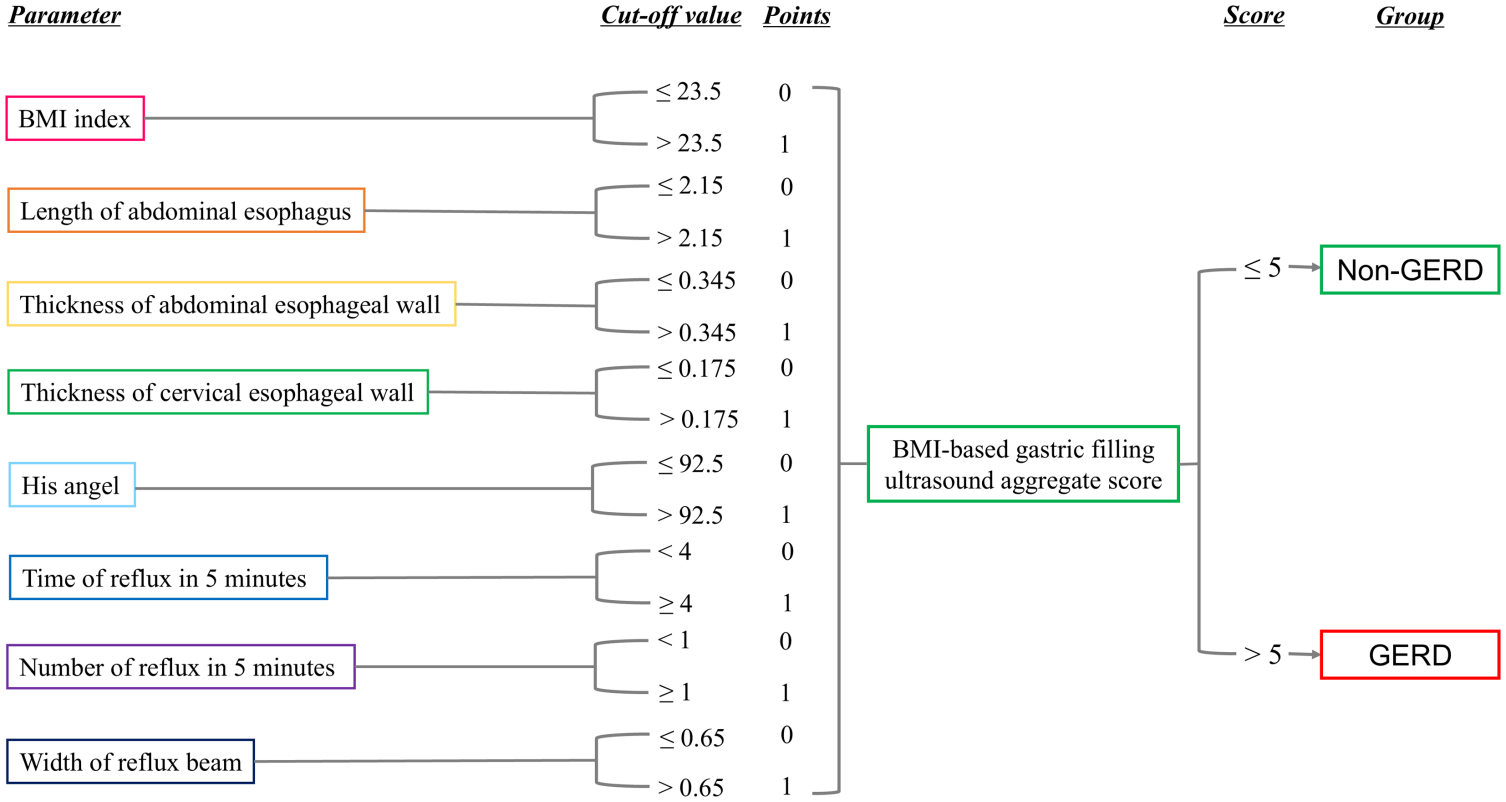
The cut-off of BMI, length of abdominal esophagus, thickness of abdominal esophageal wall, thickness of cervical esophageal wall, His angle, duration of reflux in 5 min, number of refluxes in 5 min, and width of reflux beam. The BMI-based gastric filling ultrasound aggregate score >5 was GERD.

**Figure 2:**
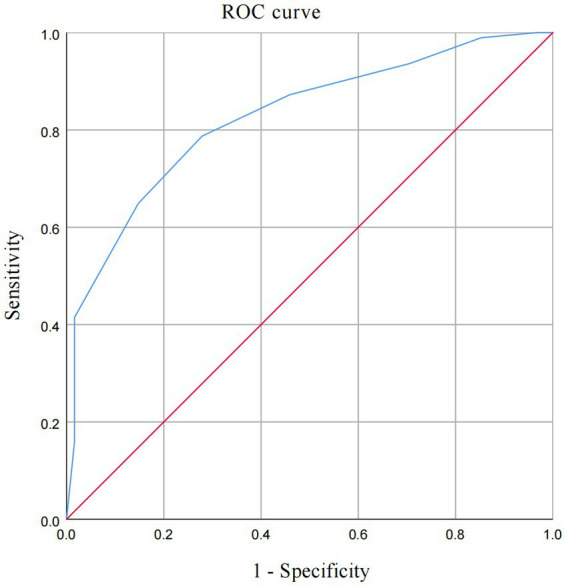
The ROC of the BMI-based gastric filling ultrasound aggregate score.

**Figure 3:**
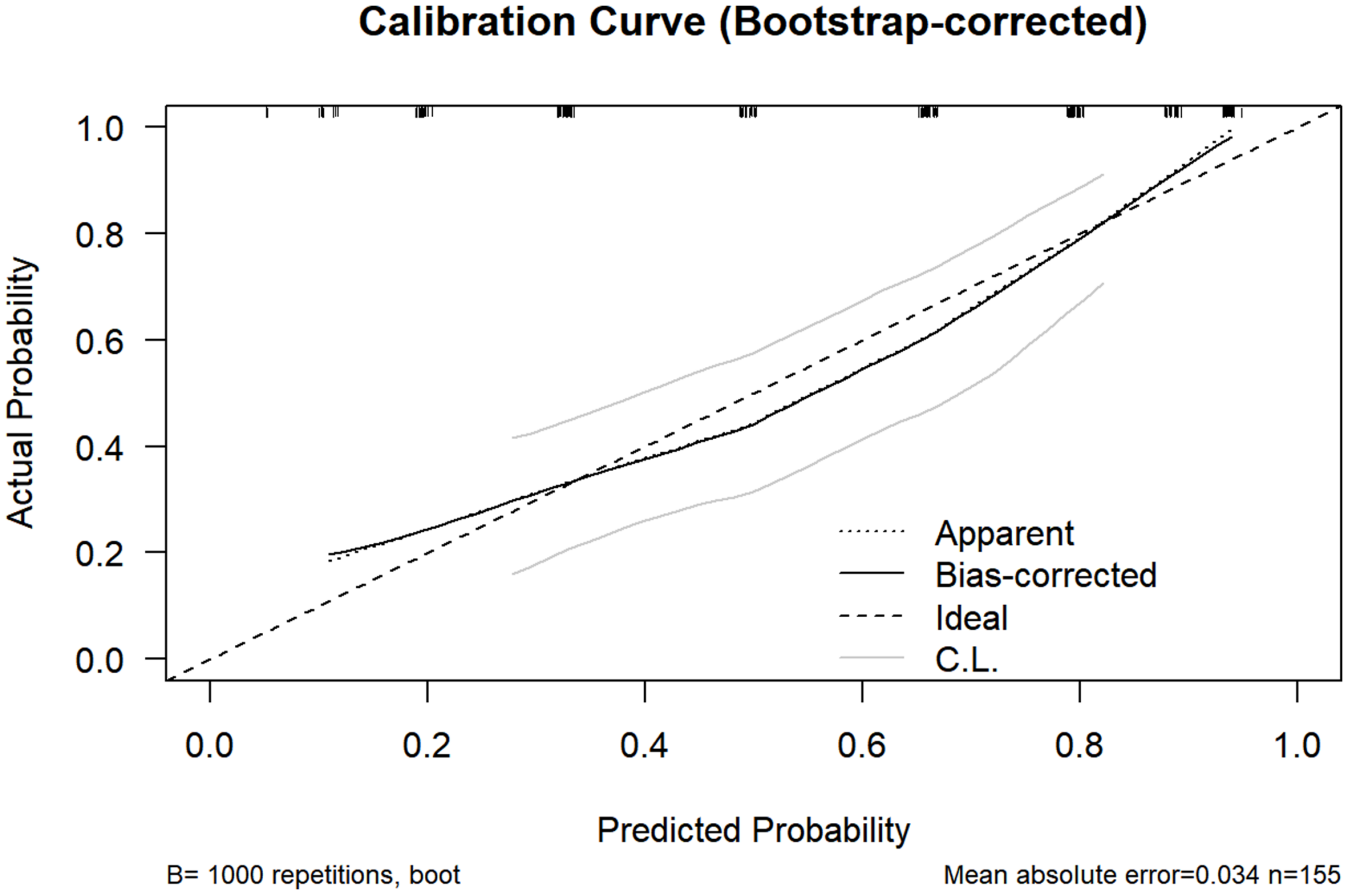
Calibration curve of the BMI-based gastric filling ultrasound scoring system for GERD diagnosis. The horizontal axis represents the predicted risk of GERD occurrence, while the vertical axis represents the actual probability of GERD occurrence. The ideal line (dashed) represents the perfect prediction of an ideal model, the bias-corrected curve (solid black) indicates the model bias correction, and the apparent line (dotted) shows the actual predictive performance. The shaded area (C.L.) denotes the confidence limits. The low mean absolute error (0.034; *n* = 155) confirms the model’s excellent calibration accuracy.

In Table 5, ROC analysis based on optimal cut-offs revealed the following diagnostic performance (AUC; sensitivity; specificity): BMI (0.678; 66.70%; 65.60%) at >23.50 kg/m^2^; abdominal esophageal length (0.462; 91.40%; 23.00%) at >2.15 cm; abdominal esophageal wall thickness (0.595; 57.00%; 68.90%) at >0.345 cm; cervical esophageal wall thickness (0.576; 73.10%; 54.10%) at >0.175 cm; His angle (0.735; 63.40%; 72.10%) at >92.50; duration of reflux in 5 min (0.739; 74.20%; 67.20%) at >4.00 s; number of reflux in 5 min (0.675; 78.50%; 50.80%) at >0.50; and width of reflux beam (0.693; 76.30%; 62.30%) at >0.65 cm. Furthermore, a comprehensive diagnostic scoring system was developed by integrating BMI and gastric filling ultrasound parameters, as detailed in the scoring algorithm in Figure 1. Each of the eight parameters—BMI, length of the abdominal esophagus, thickness of the abdominal and cervical esophageal walls, His angle, duration of reflux within 5 min, number of refluxes within 5 min, and width of the reflux beam—was assigned a score of 0 or 1 based on its optimal cut-off value. The sum of these individual scores yielded the aggregate BMI-based gastric filling ultrasound score; a total score >5 indicated GERD. The sensitivity and specificity values were 78.70 and 72.10%, respectively, as shown in Table 5. The overall diagnostic performance of this aggregate score was evaluated using ROC analysis. The score demonstrated excellent discriminative ability, with an AUC of 0.826 (Figure 2). The calibration curve, corrected for overfitting via bootstrapping (B = 1,000 repetitions), indicated good agreement between the model’s predicted probabilities and the observed outcomes, with a mean absolute error of 0.034 (n = 155), as shown in Figure 3.

**Table 5:** ROC curve analysis of BMI-based gastric filling ultrasound aggregate score and its component parameters.

		95% confidence interval (CI)					
Test result variable	AUC	Lower CI	Upper CI	Cut-off	Youden’s index	Sensitivity	Specificity
BMI index (kg/m^2^)	0.678	0.592	0.765	23.50	0.323	0.667	0.656
Length of abdominal esophagus (cm)	0.462	0.364	0.559	2.15	0.144	0.914	0.230
Thickness of abdominal esophageal wall (cm)	0.595	0.499	0.691	0.345	0.259	0.570	0.689
Thickness of cervical esophageal wall (cm)	0.576	0.482	0.671	0.175	0.190	0.731	0.541
His angle (°)	0.735	0.656	0.814	92.50	0.355	0.634	0.721
Duration of reflux in 5 min	0.739	0.661	0.817	4.00	0.414	0.742	0.672
Number of refluxes in 5 min	0.675	0.588	0.763	0.50	0.293	0.785	0.508
Width of reflux beam (cm)	0.693	0.607	0.779	0.650	0.386	0.763	0.623
BMI-based gastric filling ultrasound aggregate score	0.826	0.761	0.891	4.50	0.508	0.787	0.721

**Figure 4:**
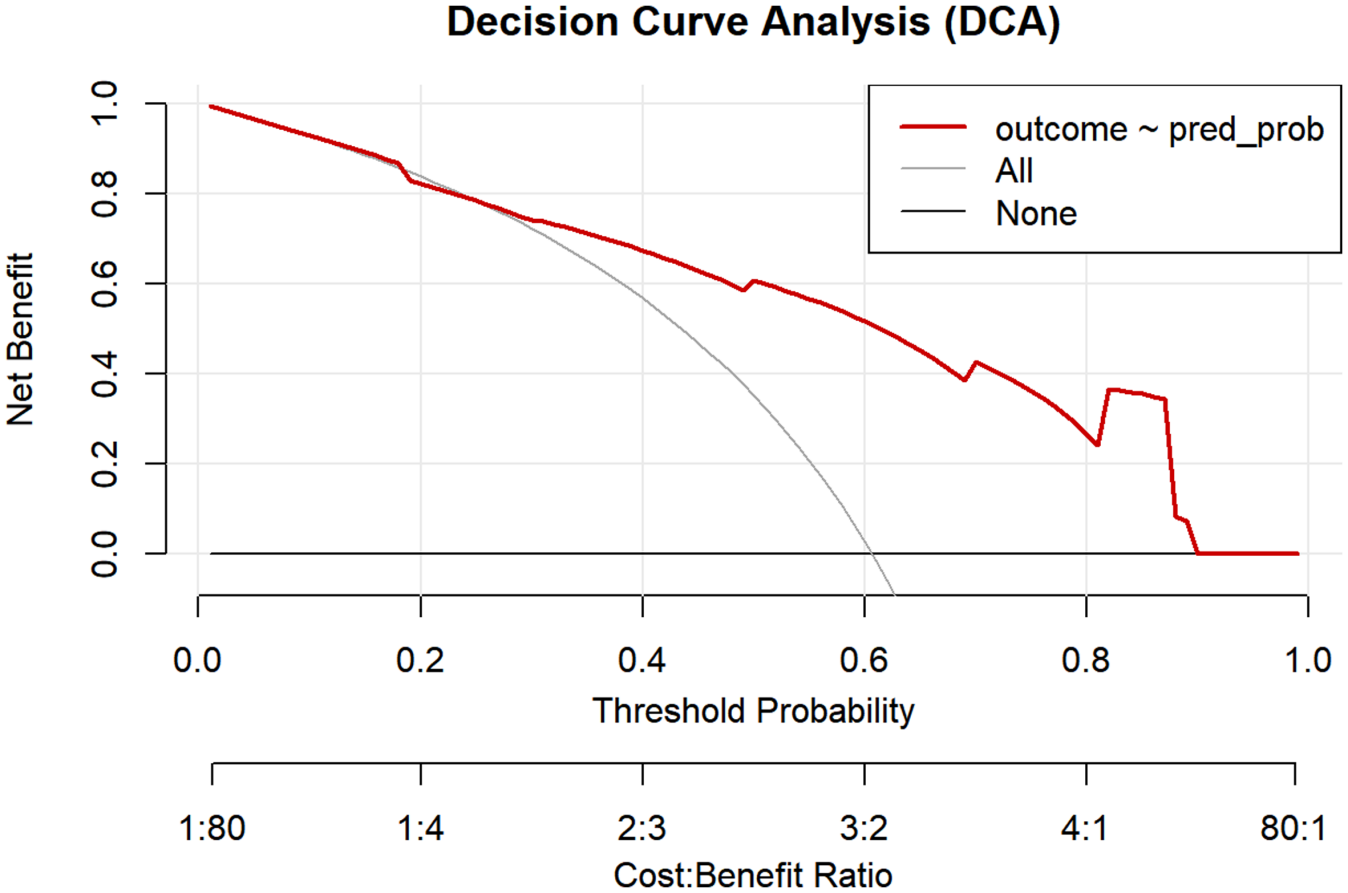
Decision curve analysis (DCA) of the BMI-based gastric filling ultrasound scoring model for GERD diagnosis. DCA curves demonstrating the maximum benefit range of the BMI-based gastric filling ultrasound scoring model. The horizontal axis represents the threshold probability, and the vertical axis represents the net benefit. The model (red line) yields a higher net benefit than the treat-all (gray line) or treat-none (black line) strategies across a threshold probability range of approximately 10–65%.

DCA was performed to evaluate the clinical utility of the BMI-based gastric filling ultrasound scoring model by quantifying its net benefit across a range of threshold probabilities, as shown in Figure 4. The net benefit of adopting the model was compared with the strategies of intervening in all patients and intervening in none across the continuum of threshold probabilities (0.0–1.0). The model provided a higher net benefit than both the treat-all and treat-none strategies across a wide range of clinically relevant threshold probabilities, approximately from 10 to 65%. This indicates that using the model to guide clinical decisions would be beneficial when a clinician’s threshold probability for intervention lies within this range. Beyond the upper limit of this range, the model’s net benefit converged with that of the “none” strategy.

As shown in Table 6, compared with 24-h pH impedance monitoring as the reference, the sensitivity of BMI-based gastric filling ultrasound was 83.75%, the specificity was 65.33%, the PPV was 72.04%, the NPV was 79.03%, and the accuracy was 74.84%. The observed agreement was 0.75, the expected agreement was 0.50, and the kappa was 0.494 (*p* < 0.001). Compared to EGD as reference, in Table 7, the sensitivity of BMI-based gastric filling ultrasound was 75.81%, the specificity was 50.54%, the PPV was 50.54%, the NPV was 75.81%, and the accuracy was 60.65%. The observed agreement was 0.61, the expected agreement was 0.48, and the kappa was 0.243 (*p* = 0.001).

**Table 6:** Comparison of the results of 24-h pH monitoring (reference standard of diagnosis) and BMI-based gastric filling ultrasound aggregate score.

Method	24-h pH monitoring – pathological	24-h pH monitoring – normal	Total	*p*-value	Kappa index
Aggregate score – pathological	67	26	93	<0.001	0.494
Aggregate score – normal	13	49	62		
Total	80	75	155		

**Table 7:** Comparison of the results of EGD (reference standard of diagnosis) and BMI-based gastric filling ultrasound aggregate score.

Method	Gastroscope – pathological	Gastroscope – normal	Total	*p*-value	Kappa index
Aggregate score – pathological	47	46	93	0.001	0.243
Aggregate score – normal	15	47	62		
Total	62	93	155		

## Discussion

Our study, for the first time, introduces a novel, non-invasive diagnostic strategy for GERD by integrating BMI with dynamic gastric filling ultrasound, a technique that enables real-time visualization of gastroesophageal anatomy and reflux events. The resulting BMI-based gastric filling ultrasound aggregate score achieved an AUC of 0.826 and demonstrated moderate agreement (*k* = 0.494). The model demonstrates superior accuracy in differentiating GERD from non-GERD and good performance in clinical utility. It supports its potential as an accessible, well-tolerated adjunct, especially for patients who cannot tolerate catheter-based monitoring or EGD. The pathogenesis of GERD is complex and involves changes in reflux exposure, epithelial resistance, and visceral sensitivity. The development of reflux disease results from a compromised anti-reflux barrier and diminished esophageal capacity to clear and neutralize refluxed material (23). GERD has complex clinical symptoms and lacks specificity. If the diagnosis is based solely on the patient’s clinical symptoms, it is difficult to accurately distinguish pathological from physiological gastroesophageal reflux. In recent years, socioeconomic development has been accompanied by an aging population and the prevalence of unhealthy lifestyles, which have collectively contributed to a rising incidence of GERD. Current trajectories suggest continued escalation in GERD prevalence with changes in people’s lifestyle and dietary patterns (24). Clinically, the occurrence of this disease is recognized as being associated with abnormalities in the structure and function of the anti-reflux barrier, reduced esophageal acid clearance, and impairment of the esophageal mucosal barrier.

Obesity and weight gain have been reported to be risk factors for GRED (25). A meta-analysis of 20 studies confirmed a significant positive association between increased BMI and GERD in the US (26). Jacobson et al. reported that the risk of GERD symptoms occurrence increases progressively with elevated BMI, even among individuals within the normal weight range (27). Obesity is a well-established risk factor for the spectrum of gastroesophageal reflux disease, demonstrating significant associations with GERD symptoms [odds ratio (OR) = 1.73], erosive esophagitis (OR = 1.59), and Barrett’s esophagus (OR = 1.24) (3, 28). Mechanisms of obesity and gastroesophageal reflux encompass elevated abdominal pressure, delayed gastric emptying, increased frequency of transient lower esophageal sphincter relaxations (TLESRs), and reduced resting pressure of the LES (29). Contemporary physiological studies highlight the role of obesity in promoting TLESRs, a primary motor event in reflux episodes (28). Consistent with previous studies, our data showed that GERD patients had significantly higher BMI than non-GERD controls (*p* < 0.001), which also justifies its inclusion in the diagnostic model.

On the one hand, with the continuous improvement of ultrasound image resolution and diagnostic techniques, gastric filling ultrasound has gained wider patient acceptance due to its safety, convenience, and non-invasive nature (11). On the other hand, it could eliminate gas interference and enhance the diagnostic efficacy for gastroduodenal diseases by filling the gastrointestinal tract with an oral contrast agent. Gastric filling ultrasound demonstration of gastric contents entering the esophagus and dilation of the distal esophagus is unlikely to be mistaken for conditions other than reflux (14). Nonacid reflux events can also be detected. Zhang et al. showed that gastric filling ultrasound demonstrated high diagnostic concordance for peptic ulcers in children and can be used as a preliminary examination method (12). Hosokawa T et al. reported a diagnostic accuracy of 88.20% in 15 patients diagnosed by gastric filling ultrasound and a concordance rate of 95.30% in duodenal ulcers (30). Jang et al. demonstrated that color Doppler sonography could detect reflux episodes with higher sensitivity than pH monitoring alone in pediatric populations (17), suggesting ultrasound’s potential for dynamic reflux quantification. Moreover, gastric filling ultrasound enables direct visualization of the anatomical structures at the gastroesophageal junction. Clinical ultrasonography in GERD enabled the dynamic visualization of esophageal peristalsis, measurement of abdominal esophageal length, and evaluation of His angle. Dynamic gastric filling ultrasound serves as a valuable complementary modality to 24-h pH impedance monitoring. It enables a detailed assessment of key anatomical landmarks. Furthermore, real-time gastric filling ultrasound provides functional and morphological evaluations, capturing dynamic processes. This integrative approach enhances the structural, functional assessment, and pH data obtained, thereby offering a more comprehensive understanding of reflux mechanisms and pathophysiology (14, 31).

In our study, when measuring the length of the abdominal esophagus, thickness of abdominal esophageal wall, thickness of cervical esophageal wall, and His angle, it was found that the length of the abdominal esophagus in the GERD patients was shorter than that in the non-GERD patients. While the thickness of the abdominal esophageal wall, the thickness of the cervical esophageal wall, and the His angle were higher than those in the non-GERD patients, which might be associated with chronic mucosal inflammation and anatomical remodeling due to recurrent reflux exposure (15). Gomes et al. demonstrated that the length of the abdominal esophagus is fundamental in reflux studies (16). Westra revealed the ability of the ultrasonographic measurement to diagnose GERD (32). Esposito et al. demonstrated that real-time sonography readily permits visualization of the gastroesophageal junction and measurement of the abdominal esophagus (14). Under normal physiological conditions, the anatomical structures at the gastroesophageal junction function to anti-reflux role. The angle of His functions acts as a unidirectional valve mechanism to prevent reflux, while the abdominal esophagus is influenced by abdominal pressure and strengthens anti-reflux capacity by driving the wall of the tube to contraction (33, 34). However, the widening of His angle indicated a weakening of the reflux barrier, leading to increased incidence of reflux events.

Recently, the diagnosis of GERD is based on EGD and/or 24-h pH impedance monitoring (35). However, the application of 24-h pH impedance monitoring methods has limitations, including technical complexity, relatively high cost, time-consuming inspection, inability to visualize anatomical defects, and invasive procedure. The placement of a trans-nasal catheter may lead to pharyngeal discomfort, nose pain, or rhinorrhea, with some patients failing to tolerate the examination. There remained certain limitations in the diagnosis of GERD via EGD. Some patients with non-erosive reflux disease showed negative results in EGD. Moreover, endoscopic procedures are invasive examination methods. In clinical practice, despite the utility of EGD, the diagnostic approach to GERD via EGD presents notable limitations. A significant proportion of patients with non-erosive reflux disease do not exhibit mucosal breaks during standard endoscopy, leading to negative endoscopic findings in a substantial number of symptomatic individuals. Moreover, as an invasive procedure, EGD carries inherent risks, including infection, injury of the throat, and even cardiovascular and cerebrovascular accidents. Consequently, the procedure is contraindicated in certain high-risk populations, such as elderly patients, individuals with multiple comorbidities, and those with severe pulmonary infections. Moreover, follow-up management in the later stage is highly significant in GERD. Since both EGD and 24-h pH impedance monitoring are invasive procedures, for some patients, there are certain difficulties in the later follow-up, and many patients may be reluctant to undergo two examinations. Therefore, we further explored the diagnostic efficacy of gastric filling ultrasound, EGD, and 24-h pH impedance monitoring for patients with gastroesophageal reflux.

In our study, when using 24-h pH impedance monitoring as the diagnostic gold standard, as listed in Table 2, the overall diagnostic accuracy of EGD was 70.32%. There was moderate agreement, with a kappa coefficient of 0.41. This likely stems from its inability to detect patients with non-erosive reflux disease; our finding is consistent with established literature. As listed in Table 3, gastric filling ultrasound demonstrated a diagnostic accuracy of 68.39%, with a kappa coefficient of 0.36 indicating minimal agreement. Jacobson et al. reported that GERD was associated with an elevated BMI, even among individuals within the normal weight range (27). Thus, to improve diagnostic performance, we integrated BMI and gastric filling ultrasound findings, including the thickness of the abdominal esophageal wall, the His angle, the duration of reflux in 5 min, the number of refluxes in 5 min, and the width of the reflux beam. In our study, we developed a novel scoring system, namely, the BMI-based gastric filling ultrasound aggregate score. The score was calculated using the formula shown in Figure 1. The optimal critical value of the BMI-based gastric filling ultrasound was 4.50; it had the largest area under the curve (0.826) in Figure 2. The calibration curve shown in Figure 3 indicates good agreement between the predicted probabilities of the model and the observed outcomes, with a mean absolute error of 0.034. DCA was performed to evaluate the clinical utility of the BMI-based gastric filling ultrasound model. As shown in Figure 4, the model demonstrates superior accuracy and strong performance in calibration and clinical utility. The model defined scores of 0–5 as non-GERD and scores of 6–8 as GERD. As presented in Table 6, the novel combined approach significantly enhanced diagnostic accuracy to 74.84%, with a kappa coefficient of 0.494 indicating moderate agreement. Furthermore, when integrated with BMI and gastric filling ultrasound, this multimodal strategy demonstrated improved diagnostic efficacy and exhibited good predictive value.

To date, we have not considered gastric filling ultrasound as a substitute for established diagnostic methods but rather as an adjunctive diagnostic tool. It is particularly suitable for patients unable to tolerate 24-h pH impedance monitoring and EGD. This non-invasive technique offers several advantages, including excellent reproducibility, procedural simplicity, ease of use, real-time observation of anatomical structures, painlessness, safety, and time efficiency, all of which facilitate its widespread clinical adoption and suitability for follow-up management. Additionally, the relatively lower cost of this examination may help reduce the financial burden on patients with GERD.

Our study also had some limitations. First, the study was conducted at a single center. All data were obtained from a single relatively small sample of patients. Second, these patients lack follow-up data. Third, since our diagnostic accuracy study required patients to undergo three separate investigations, our cohort was recruited from a tertiary center. The willingness to participate in our study may be associated with higher health motivation or more severe symptoms. This may affect the generalizability of our findings to the broader community-based population with GERD. Future studies conducted in primary or secondary care settings are warranted to validate the applicability of the gastric filling ultrasound in a less selected patient population. Moreover, it is important to note that our study specifically enrolled patients presenting with typical esophageal symptoms (regurgitation, acid reflux, or heartburn). While we recognize that GERD can manifest with a variety of extraesophageal symptoms, such as chronic cough, asthma, and dental erosion, establishing a definitive causal link between reflux events and these symptoms is often challenging and requires a multifaceted diagnostic approach. Future studies are warranted to explore the potential utility of the BMI-based gastric filling ultrasound in the diagnostic workup of patients with suspected extraesophageal GERD syndromes. Therefore, large-scale, multicenter follow-up are warranted in the future.

## Conclusion

Gastric filling ultrasound is a dynamic imaging technique that visualizes gastroesophageal reflux in patients with GERD-like symptoms. The BMI-based gastric filling ultrasound, given its strong correlation with 24-h pH impedance monitoring, is a promising adjunct for diagnosing GERD.

## Data Availability

The original contributions presented in the study are included in the article/Supplementary material, further inquiries can be directed to the corresponding authors.

## References

[1] Durazzo M Lupi G Cicerchia F Ferro A Barutta F Beccuti G . Extra-esophageal presentation of gastroesophageal reflux disease: 2020 update. J Clin Med. (2020) 9:2559. doi: 10.3390/jcm9082559, 32784573 PMC7465150

[2] Zhang D Liu S Li Z Wang R. Global, regional and national burden of gastroesophageal reflux disease, 1990-2019: update from the GBD 2019 study. Ann Med. (2022) 54:1372–84. doi: 10.1080/07853890.2022.2074535, 35579516 PMC9122392

[3] Eusebi LH Ratnakumaran R Yuan Y Solaymani-Dodaran M Bazzoli F Ford AC. Global prevalence of, and risk factors for, gastro-oesophageal reflux symptoms: a meta-analysis. Gut. (2018) 67:430–40. doi: 10.1136/gutjnl-2016-313589, 28232473

[4] Yamasaki T Hemond C Eisa M Ganocy S Fass R. The changing epidemiology of gastroesophageal reflux disease: are patients getting younger? J Neurogastroenterol Motil. (2018) 24:559–69. doi: 10.5056/jnm18140, 30347935 PMC6175565

[5] Bruley des Varannes S Löfman HG Karlsson M Wahlqvist P Ruth M Furstnau ML . Cost and burden of gastroesophageal reflux disease among patients with persistent symptoms despite proton pump inhibitor therapy: an observational study in France. BMC Gastroenterol. (2013) 13:39. doi: 10.1186/1471-230x-13-39, 23448382 PMC3610279

[6] Gyawali CP Yadlapati R Fass R Katzka D Pandolfino J Savarino E . Updates to the modern diagnosis of GERD: Lyon consensus 2.0. Gut. (2024) 73:361–71. doi: 10.1136/gutjnl-2023-330616, 37734911 PMC10846564

[7] Yadlapati R Gyawali CP Pandolfino JE. AGA clinical practice update on the personalized approach to the evaluation and management of GERD: expert review. Clin Gastroenterol Hepatol. (2022) 20:984–994.e1. doi: 10.1016/j.cgh.2022.01.025, 35123084 PMC9838103

[8] Barcelo M Alvarez Sanchez A Garcia Sanchez R Diaz-Rubio M Rey E. Weight gain and somatization are associated with the onset of gastroesophageal reflux diseases: results of two 5-year follow-up studies. J Clin Gastroenterol. (2016) 50:202–7. doi: 10.1097/mcg.0000000000000364, 26084009

[9] Sweis R Fox M Anggiansah R Anggiansah A Basavaraju K Canavan R . Patient acceptance and clinical impact of bravo monitoring in patients with previous failed catheter-based studies. Aliment Pharmacol Ther. (2009) 29:669–76. doi: 10.1111/j.1365-2036.2008.03923.x, 19183144

[10] Zhou SF Yin JB Yang H Zhong J An P. Application value of stomach filling ultrasonography and intravenous contrast agents in diagnosis of advanced gastric cancer. Eur Rev Med Pharmacol Sci. (2016) 20:3206–10. 27466993

[11] Nielsen MB Søgaard SB Bech Andersen S Skjoldbye B Hansen KL Rafaelsen S . Highlights of the development in ultrasound during the last 70 years: a historical review. Acta Radiol. (2021) 62:1499–514. doi: 10.1177/02841851211050859, 34791887

[12] Zhang YH Xu ZH Ni SS Luo HX. Gastrointestinal contrast-enhanced ultrasonography for diagnosis and treatment of peptic ulcer in children. World J Gastroenterol. (2024) 30:705–13. doi: 10.3748/wjg.v30.i7.705, 38515948 PMC10950618

[13] Wang J Yang Y Ding L Cui J Ye H Ruan H . Diagnostic value of contrast-enhanced ultrasonography in preoperative Borrmann classification of gastric cancer. Zhonghua Wei Chang Wai Ke Za Zhi. (2014) 17:254–7. 24671814

[14] Minella R Minelli R Rossi E Cremone G Tozzi A. Gastroesophageal and gastric ultrasound in children: the state of the art. J Ultrasound. (2021) 24:11–4. doi: 10.1007/s40477-020-00471-w, 32361921 PMC7925781

[15] Ribolsi M Savarino E. Exploring the association between esophageal mucosal inflammation, impaired motility, and GERD severity. Neurogastroenterol Motil. (2021) 33:e14211. doi: 10.1111/nmo.14211, 34431169

[16] Gomes H Hornoy P Liehn JC. Ultrasonography and gastric emptying in children: validation of a sonographic method and determination of physiological and pathological patterns. Pediatr Radiol. (2003) 33:522–9. doi: 10.1007/s00247-003-0954-1, 12811435

[17] Jang HS Lee JS Lim GY Choi BG Choi GH Park SH. Correlation of color Doppler sonographic findings with pH measurements in gastroesophageal reflux in children. J Clin Ultrasound. (2001) 29:212–7. doi: 10.1002/jcu.1022, 11323775

[18] Maleki I Borhani S Moosazadeh M Alizadeh-Navaei R. Prevalence and risk factors of gastroesophageal reflux disease symptoms in Mazandaran, north of Iran: a Tabari cohort study. Caspian J Intern Med. (2024) 15:280–6. doi: 10.22088/cjim.15.2.280, 38807738 PMC11129062

[19] Chowdhury SD George G Ramakrishna K Ramadass B Pugazhendhi S Mechenro J . Prevalence and factors associated with gastroesophageal reflux disease in southern India: a community-based study. Indian J Gastroenterol. (2019) 38:77–82. doi: 10.1007/s12664-018-00931-6, 30790137

[20] Johnson LF DeMeester TR. Development of the 24-hour intraesophageal pH monitoring composite scoring system. J Clin Gastroenterol. (1986) 8 Suppl 1:52–8. doi: 10.1097/00004836-198606001-00008, 3734377

[21] Vakil N van Zanten SV Kahrilas P Dent J Jones R. The Montreal definition and classification of gastroesophageal reflux disease: a global evidence-based consensus. Am J Gastroenterol. (2006) 101:1900–20. doi: 10.1111/j.1572-0241.2006.00630.x 16928254

[22] Fazzini B Märkl T Costas C Blobner M Schaller SJ Prowle J . The rate and assessment of muscle wasting during critical illness: a systematic review and meta-analysis. Crit Care. (2023) 27:2. doi: 10.1186/s13054-022-04253-0, 36597123 PMC9808763

[23] Tack J Pandolfino JE. Pathophysiology of gastroesophageal reflux disease. Gastroenterology. (2018) 154:277–88. doi: 10.1053/j.gastro.2017.09.047, 29037470

[24] Argyrou A Legaki E Koutserimpas C Gazouli M Papaconstantinou I Gkiokas G . Risk factors for gastroesophageal reflux disease and analysis of genetic contributors. World J Clin Cases. (2018) 6:176–82. doi: 10.12998/wjcc.v6.i8.176, 30148145 PMC6107529

[25] Viazis N Karamanolis GP Anastasiou J Keyoglou A Vlachogiannakos J Ladas SD . Refractory GERD: increased body mass index is associated with persisting acid exposure but not hypersensitive esophagus or functional heartburn. Eur J Gastroenterol Hepatol. (2013) 25:1450–5. doi: 10.1097/MEG.0b013e328365d2a8 24047861

[26] Hampel H Abraham NS El-Serag HB. Meta-analysis: obesity and the risk for gastroesophageal reflux disease and its complications. Ann Intern Med. (2005) 143:199–211. doi: 10.7326/0003-4819-143-3-200508020-00006, 16061918

[27] Jacobson BC Somers SC Fuchs CS Kelly CP Camargo CA Jr. Body-mass index and symptoms of gastroesophageal reflux in women. N Engl J Med. (2006) 354:2340–8. doi: 10.1056/NEJMoa054391, 16738270 PMC2782772

[28] Richter JE Rubenstein JH. Presentation and epidemiology of gastroesophageal reflux disease. Gastroenterology. (2018) 154:267–76. doi: 10.1053/j.gastro.2017.07.045, 28780072 PMC5797499

[29] Anand G Katz PO. Gastroesophageal reflux disease and obesity. Gastroenterol Clin N Am. (2010) 39:39–46. doi: 10.1016/j.gtc.2009.12.002, 20202577

[30] Hosokawa T Tanami Y Sato Y Hara T Iwama I Ishimaru T . Diagnostic accuracy of ultrasound for detecting gastric or duodenal ulcers in pediatric patients. J Ultrasound Med. (2022) 41:457–69. doi: 10.1002/jum.15727 33876858

[31] Savino A Cecamore C Matronola MF Verrotti A Mohn A Chiarelli F . US in the diagnosis of gastroesophageal reflux in children. Pediatr Radiol. (2012) 42:515–24. doi: 10.1007/s00247-012-2344-z, 22402830

[32] Westra SJ Derkx HH Taminiau JA. Symptomatic gastroesophageal reflux: diagnosis with ultrasound. J Pediatr Gastroenterol Nutr. (1994) 19:58–64. doi: 10.1097/00005176-199407000-00009 7965478

[33] Gu L Chen B Du N Fu R Huang X Mao F . Relationship between bariatric surgery and gastroesophageal reflux disease: a systematic review and Meta-analysis. Obes Surg. (2019) 29:4105–13. doi: 10.1007/s11695-019-04218-3, 31630324

[34] Elsheaita A El-Bially MA Shamseya MM Ahmed SS Madkour MA Shamseya AM . Seattle protocol vs narrow band imaging guided biopsy in screening of Barrett's esophagus in gastroesophageal reflux disease patients. Medicine (Baltimore). (2020) 99:e19261. doi: 10.1097/md.0000000000019261, 32080134 PMC7034706

[35] Lombo-Moreno C La Rotta D Pardo-Ortiz M Avila FA Cañadas RA Muñoz Ó . Diagnostic accuracy of mean nocturnal basal impedance and other complementary tests for the diagnosis of gastroesophageal reflux disease according to the new Lyon criteria. Ther Adv Gastroenterol. (2025) 18:17562848251340495. doi: 10.1177/17562848251340495, 40417713 PMC12099136

