# Supplementary material for: Diagnostic accuracy of gastric filling ultrasound combined with BMI for gastroesophageal reflux disease
Source: Front Med (Lausanne). 2026 Feb 19;13:1727623. doi: 10.3389/fmed.2026.1727623 (PMC12960124; doi:10.3389/fmed.2026.1727623)
Supplement: Supplementary file 1 [file Data_Sheet_1.docx]

Supplement table 1. The correlation between ultrasound parameters and 24-hour PH impedance-monitoring parameters

| **Spearman correlation coefficient(P****-value)** | | **24-hour PH monitoring parameters** | | |
| --- | --- | --- | --- | --- |
|  |  | **Number of refluxes** | **Acid exposure time (AET)** | **DeMeester score** |
| **Gastric filling ultrasound parameters** | Length of abdominal esophagus (cm) | -0.164  (0.165) | -0.159  (0.241) | -0.091  (0.405) |
|  | Thickness of abdominal esophageal wall (cm) | -0.005  (0.968) | 0.213  (0.114) | 0.090  (0.408) |
|  | Thickness of cervical esophageal wall (cm) | 0.302  **(0.009)** | 0.331  **(0.013)** | 0.236  **(0.029)** |
|  | His angle (°) | 0.181  (0.126) | 0.198  (0.143) | 0.217  **(0.044)** |
|  | Duration of reflux in 5 minutes(s) | 0.100  (0.399) | 0.104  (0.444) | 0.069  (0.525) |
|  | Number of refluxes in 5 minutes | 0.044  (0.711) | 0.030  (0.827) | -0.037  (0.734) |
|  | Width of reflux beam(cm) | 0.078  (0.514) | 0.065  (0.634) | 0.054  (0.622) |
